# Modern Analytical Methods for the Analysis of Pesticides in Grapes: A Review

**DOI:** 10.3390/foods11111623

**Published:** 2022-05-31

**Authors:** Yerkanat Syrgabek, Mereke Alimzhanova

**Affiliations:** 1 Center of Physical-Chemical Methods of Research and Analysis, Al-Farabi Kazakh National University, Tole bi 96a, Almaty 050012, Kazakhstan; serkanat96@gmail.com; 2 Faculty of Physics and Technology, Al-Farabi Kazakh National University, 71 al-Farabi Ave., Almaty 050040, Kazakhstan

**Keywords:** pesticides, grape, residues, extraction, detection

## Abstract

Currently, research on the determination of pesticides in food products is very popular. Information obtained from research conducted so far mainly concerns the development of a methodology to determine the content of pesticides in food products. However, they do not describe the content of the pesticide used in viticulture in the resulting product. Over the past decade, this study has examined analytical methodologies for assessing pesticide residues in grapes. Scopus, Web of Science, Science Direct, PubMed, and Springer databases were searched for relevant publications. The phrases “pesticides” and “grapes” and their combinations were used to search for articles. The titles and annotations of the extracted articles have been read and studied to ensure that they meet the review criteria. The selected articles were used to compile a systematic review based on scientific research and reliable sources. The need to study the detection of pesticide residues in grapes using advanced analytical methods is confirmed by our systematic review. This review also highlights modern methods of sample preparation, such as QuEChERS, SPME, PLE, dLLME, and ADLL-ME, as well as the most used methods of separation and identification of pesticides in grapes. An overview of the countries where residual grape pesticide amounts are most studied is presented, along with the data on commonly used pesticides to control pests and diseases in grape cultivation. Finally, future possibilities and trends in the analysis of pesticide residues in grapes are discussed by various analytical methods.

## 1. Introduction

Grapes are increasingly widely used, both in the form of the grape and in its by-products. Due to its excellent nutritional characteristics, grape farming and the production of by-products are significant. Every year, the production of grapes and grape-based goods such as wine, jam, juice, vinegar, raisins, and grape seed oil increases [1].

Food quality has become an important and very serious issue due to the increasing use of pesticides [2]. When grapes are grown, pesticides are used to combat potential pests and diseases. There is a severe danger of vine disease at all stages of ripening with different types of fungi [3]. Furthermore, during ripening, in addition to diseases and fungi, grapes can be negatively affected by various insects [4]. More pesticides and insecticides are used to combat unwanted pests of grapes. Sometimes, pesticides are misused in grape cultivation, thus exceeding the allowable level of pesticide residues [5]. Pesticide residues in grapes can damage the environment, affect the quality of grapes and their processed products, and even affect human health [6].

An analysis of the literature review showed that pesticides with 33 main active ingredients are used in the fight against insects and diseases of grapes (Table 1). Data on grape pesticide use and limits of acceptable (LAC) concentrations were obtained from the European Commission [7]; the lowest limit of acceptable concentration is 0.01 mg/kg.

In order to evaluate low pesticide levels in grapes, sensitive, highly selective, and accurate analytical techniques are required due to the increasing pesticide usage each year. Different instrumental approaches are used to assess and identify pesticides in grapes and their processed products. In the scientific literature, high-performance liquid chromatography (HPLC), as well as gas and liquid chromatography (GC, LC), are the most commonly used techniques [8]. New research released in 2019 suggests table grapes are contaminated with 96 different pesticides. The authors of identified and quantified 96 pesticides residues by gas and liquid chromatography in conjunction with tandem mass spectrometry per grape sample [9]. In another article that was published in 2020, the authors investigated pesticides such as penconazole, hexaconazole, diazinon, ethion, and phosalone by gas chromatograph with mass spectrometric detection methods [10]. The authors [11] quantitatively determined pesticides using liquid chromatography in combination with tandem mass spectrometry. Extraction and sample preparation methods are also important for determination of pesticides in different plant samples. The QuEChERS method is one of the popular sample preparation methods.

In addition to the known analytical methods, researchers are developing and testing their own methods for determining pesticide residues. Even when using the same analytical method to determine pesticide residues, different equipment and sample preparation methods can be selected. There is a manual [12] that gives laboratories a free choice of analytical methods and encourages the development of new methods for determining pesticide residues.

**Figure 1:**
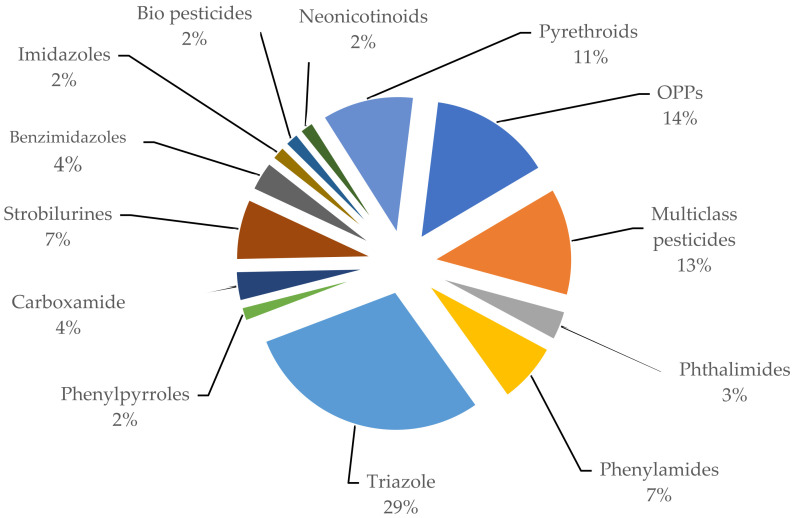
Classes of pesticides are most commonly used to control pests and diseases at different stages of grape cultivation.

Analytical methods used for determining pesticide residues in grapes over the last decade are discussed in this review. The most often used classes of pesticides in grapes from 2015 to 2021 years are illustrated in Figure 1.

**Table 1:** List of pesticides most commonly used to control pests and diseases at different stages of grape cultivation.

Number	Pesticides	Class of Pesticides	Application	LAC (mg/kg)	References
1	Abamectin	Avermectins, Biological pesticides	Rape and grape	0.01	[13,14]
2	Ametrine	Other substances	Grapes		[14,15]
3	Boscalid	Contact fungicide from the carboxamide class	Against diseases of grapes (grey rot), against diseases of grapes (oidium)	5	[16,17]
4	Captan	Phthalimides	Cotton, grapes, apple tree, rapeseed	0.03	[18]
5	Carbendazim	Benzimidazoles	Grapes	0.3	[14,19,20,21,22]
6	Chlorpyrifos	Organophosphates	Cotton, sugar beet, apple, peach, potato, hops, alfalfa. Areas filled with locusts. Melons, grapes, onions, rapeseed, corn, sunflower	0.01	[15,22,23]
7	Cypermethrin	Pyrethroids	Cotton, sugar beet, apple, peach, potato, hops, alfalfa. Areas filled with locusts. Melons, grapes, onions, rapeseed, corn, sunflower	0.5	[18]
8	Cypermethrin-alpha	Pyrethroids	Spring wheat, locust filling, rapeseed, grapes, apple tree, sugar beet, potatoes, cotton	0.5	[18]
9	Cyprodinil	Aminopyrimidine	Grapes	3	[17,24]
10	Dichlorobenzamide	Benzamides	Grapes, wine, and raisins		[16]
11	Dimethomorph	Other substances	Grapes	3	[20,22,25]
12	Diniconazole	Triazoles	Grapes	0.01	[15,25,26,27]
13	Ethion	Organothiophosphate	Grapes	0.01	[10,18,25]
14	Fenitrothion	Organophosphorus	Grapes	0.01	[23,25]
15	Fenthion	Organophosphorus	Grapes	0.01	[14,23]
16	Fludioxonil	Benzodioxoles	Grapes	5	[19,24]
17	Fluopicolide	Other substances	Grape or soil sample	2	[14,16,28]
18	Folpet	Phthalimide	Meadow, vineyards, tomato, cucumbers	6	[18,24,29]
19	Hexaconazole	Triazole	Grapes	0.01	[10,19,22]
20	Lambda-cyhalothrin	Pyrethroids	Grapes	0.08	[18]
21	Metalaxyl	Other substances	Grapes	2	[16,20,24,29]
22	Methomyl	Carbamate	Appletree, apricot, grapes, tomatoes, onions, cabbage, cucumbers, cotton	0.01	[18]
23	Oxadiazon	Aromatic pesticide	Grape	0.01	[15,26]
24	Penconazole	Triazoles	Grapes	0.5	[15,24,26]
25	Phosalone	Organophosphorus	Grapes	0.01	[10,22]
26	Picoxystrobin	Strobilurines	Grapes, wine, and raisins	0.01	[16]
27	Prochloraz	Imidazoles	Cabbage, apple, kiwi, pear, grape	0.03	[18,19]
28	Procymidone	Other substances	Grapes	0.01	[18,24]
29	Propiconazole	Triazole	To combat diseases of grain, grapevine	0.01	[17,20,25]
30	Pyraclostrobin	Strobilurines	Grapes	0.3	[16,17,30,31]
31	Pyrimethanil	Aminopyrimidines	Lettuce garlic shoot, yam, celery, carrot, pepper, chives, cowpea, tomato, spinach, cabbage, apple, kiwi, pear, grape	5	[17,20]
32	Tebuconazole	Third generation Triazole	For the treatment of grain seeds in the fight against phytopathogens transmitted with seeds, grape.	0.5	[15,19,20,21,26]
33	Thiophanate-methyl	Thioureas	Table grape	0.1	[19,20,21]

According to Figure 1, pesticides of the triazole class are used most often (~29%) in the processing of grapes at different stages of cultivation. This is explained by the fact that pesticides of this class are chemicals that effectively control and destroy harmful microorganisms and are also fungicides for a wide range of uses with low toxicity [20,25]. After the triazole class fungicides, organophosphate pesticides are the next commonly known and used (~14%), which effectively fight against the pests of the grapes [32,33,34,35]. A widespread pesticide used for the cultivation of grape is multiclass pesticides, which involve 13% of other pesticides with different biological activities such as fungicides, acaricides, insecticides, herbicides, and plant growth regulators [9,22]. The choice of the use of different pesticides, depends on many factors. Environmental conditions, such as sunlight, temperature and humidity, play an essential role in the kinetic and dynamic behaviors of pesticides [14,17,18]. The use of separate classes of pesticides helps to solve problems of various kinds; for example, one of the commonly used pesticides is pyrethroids [18,25,33]. Pyrethroids are a synthetic class of pesticides derived from natural chrysanthemum esters. Like other pesticides, they can accumulate and spread through all the links of food cultivation and, accordingly, pollute the daily diet of humans [32].

Given their importance in maintaining the effectiveness of products, pesticide residues in grapes and their processed products should be carefully monitored. Various techniques for determining pesticide residues have been developed in this area. A thorough assessment of the literature was conducted in search engines such as Google Scholar, PubMed, Scopus and Web of Science to conduct the research. “Pesticide residues”, “extraction procedures”, “detection methods”, and “grapes” were used as search terms for the literature study.

This review describes the recent analytical methods of the determination pesticides residues in grapes and future advantages of application. The review will provide practical assistance for analytical laboratories in the field of pesticide analysis in grapes and for regulators in monitoring food quality and safety.

## 2. Sample Preparation Methods

Pesticide residues in grapes and their processed products were determined using a range of sampling and extraction methods, as indicated in Table 2. There is no universal method of extraction. When evaluating the residual quantities of pesticides in grapes, authors use various extraction procedures depending on the pesticide and grape properties.

**Figure 2:**
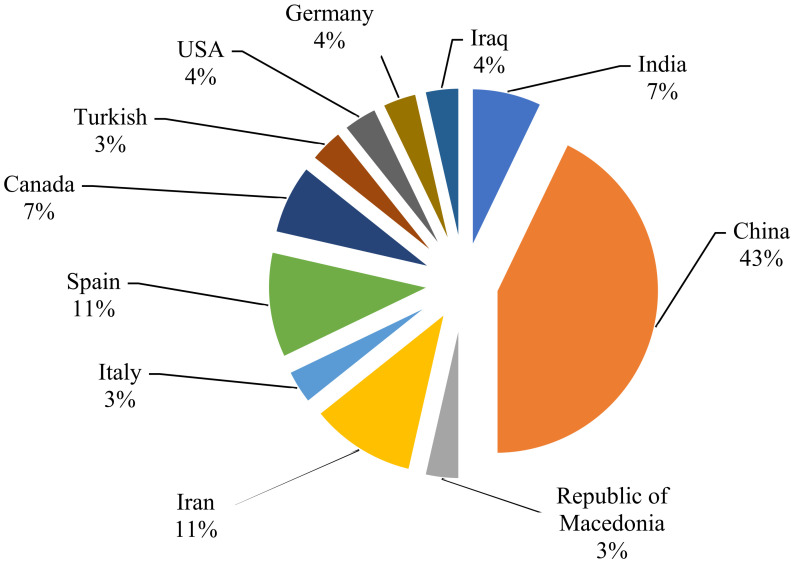
An overview of the countries that determined the residual amounts of pesticides in grapes in 2015–2021.

The top grape-producing countries are China, Italy, USA and Spain [41]. The above data in Figure 2 show that China is one of the leading countries having studied and determined pesticide residues in grapes. The reason is that, over the past decade, the use of pesticides has increased worldwide due to an ever-growing population and rapid urbanization [42].

Spain has the largest vineyard area in the world. The climate in Spain is highly diverse, and many “microclimates” can be found throughout the country, each of which has a different effect on the cultivation of different grape varieties [39]. Since many grapes are grown in Spain, there is a need to strictly verify this product at all stages of cultivation and production of secondary products.

Currently, pesticides play an important role in increasing agricultural productivity, particularly grape yields. Even though grapes are grown in separate and specialized places for cultivation, most pesticides used to control pests and various diseases of grapes have a negative impact on the human body. Therefore, there are serious concerns about the excessive use of pesticides [24].

Since different countries have specific climatic conditions and methods of growing grapes, their own methods of sample preparation and determination of target analytes are used. Researchers from different countries explore the methods most suitable for their place of residence. For example, the authors of the Indies [23] write that inappropriate farming methods during the use of pesticides led to higher contamination of grapes. According to this, studies on the effect of grape pesticides in countries such as Italy and France are significantly fewer compared to other countries. This is most likely since, in these countries, the cultivation of grapes is well developed and retains its status with a lot of local regulatory authorities.

Countries such as India, Turkey and Iran are the top ten grape producing countries, and scientists from these countries are also actively studying pesticide residues [15,18,22].

### 2.1. Quick, Easy, Cheap, Effective, Rugged, and Safe Method (QuEChERS)

This method has become widely used because of its micro-scale extraction procedure, which requires less time and less organic solvent [15]. Usually, this method involves acetonitrile in the extraction process for effective extraction. Acetonitrile mixes well with water and can be separated from salt before final purification [16].

In [43], a study was conducted using the QuEChERS method without a purification process. Multiclass pesticides with detection and quantification limits of 5 µg/kg and 10 µg/kg, respectively, were successfully detected in this method. The method was simple and provided excellent extraction (73–111%) with an RSD value of ≤19.7%. In addition, the authors concluded that the matrix effect is within the limits of acceptable values.

Currently, a method with modified dissolution conditions, such as acetonitrile and ethyl acetate, is used, which is more suitable for detection by gas chromatography [44,45] and liquid chromatography [46]. Over the past decade, the approach to the QuEChERS method has surpassed significant changes. This method is often used due to its efficiency in extracting a wide range of analytes, good flexibility, and the smallest volume of solvent. The results obtained during the study show that the QuEChERS method is more effective compared to other methods.

### 2.2. Solid-Phase Extraction (SPE)

The SPE method is the most used due to its simplicity, speed and ability to process a large volume of samples. Furthermore, this method uses a wide range of cartridges, such as C8, C18, etc., for pretreatment and determination of pesticide residues in vegetables and fruits.

The classical sorbents used in the SPE method retain the analyzed substances because of non-selective hydrophobic reactions. This leads to the joint extraction of interfering elements and low cleaning efficiency. For this reason, other complex pre-cleaning procedures are required. In the study conducted by [32], a variant of a molecular imprinted polymer (MIP) was used as a high-quality sorbent. Having stable physico-chemical characteristics, MIP has significant limitations in the analysis of organophosphate pesticides due to multiple pesticide residues.

Residual evaluation is mainly carried out with typical sorbents, such as graphite carbon black and primary secondary amine (PSA) [22]. In some cases, sorbents (PSA-B-C18) are used together in the purification process to increase the sensitivity of the method. The choice of different solvents depends on the molecular characteristics (ionic and nonionic) of the analyzed pesticides. Commonly used solvents include toluene, hexane, acetic acid, acetone, dichloromethane, ethyl acetate, methanol and acetonitrile. Various articles state that the SPE method is a fast and effective method of analyzing pesticides; thus, it provides good separation and recovery from complex matrices. Additionally, this method causes cartridge clogging with suspended sample particles and has the possibility of low extraction when sorbents interact with the analyzed substances.

### 2.3. Solid-Phase Micro-Extraction (SPME)

Solid-phase microextraction is a method of sample preparation with features such as ease of use, portable, fast and solvent-free. The method is based on the separation of the analyzed substances between the phases immobilized on the fiber.

Using an internal standard for each target connection is economically and practically inefficient for multicomponent connections. In [17], the main goal was to study a small number of different chemical internal standards for determining target analytes. The CBS-MS/MS method was successfully used, which made it possible to compare target analytes with internal standards using the internal standards panel. Solid particles are often found in diluted multicomponent samples. The SPME method has several advantages.

Scientists [47] have described the process of obtaining fiber by carbonation. The process of obtaining fibers from the SPME method turned out to be effective in the ability to extract target analytes, the results of which are indicated in this article. The authors indicated that the purpose of fiber modifications was to enhance the adsorption of MOF deposition, which was previously challenging. One of the important parameters for SPME coverage is stability. To study stability, the fiber was soaked in different solvents under different conditions and then used to extract eight peritroids. The results showed that the stability remained unchanged and has good extraction ability.

**Figure 3:**
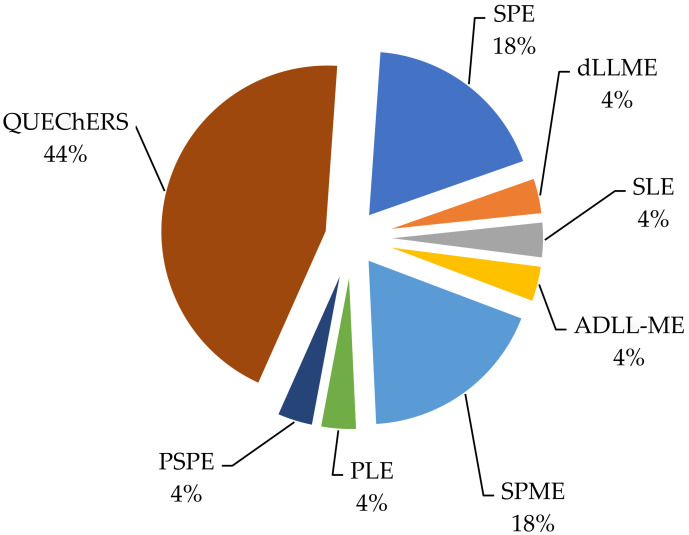
The most used methods of sample preparation and extraction in the determination of pesticides in grapes.

Sample preparation methods such as SPE and LLE are widely used to determine fungicides in grapes. These sample preparation methods consume a lot of organic solvents and are labor-intensive [19]. One of the reasons the SPE method is rarely used now is that classical sorbents (C8, C18) retain the analyzed substances by a non-selective hydrophobic reaction, which leads to partial joint extraction of interfering substances [32]. In the article [48], two sample preparation methods are compared, SPME and QuEChERS. This article shows that between the two methods, SPME is more environmentally friendly. The authors attribute this to the fact that SPME is performed at the microscale, while QuEChERS is at the macroscale and requires extraction solvents and significant additional processing. Despite this, the QuEChERS sample preparation method in Figure 3 remains the most popular among its analogues and occupies more than half of the methods.

### 2.4. Other Sample Preparation Methods

During sample preparation, it is necessary to consider the physico-chemical properties of the analyzed substance, mainly the polarity of the pesticide. The evolution of extraction methods combined with parallel improvement of analytical methods has reduced the complexity of sample processing and increased the accuracy of the analysis.

In addition to the classical sample preparation methods, other methods have often been used recently. For example, in [49], 19 pesticides were quantified by trace amounts. The dispersive liquid-liquid microextraction (DLLME) method has proven to be an excellent alternative extraction method for determining pesticides in complex matrices.

Because it is necessary to consider the Physico-chemical properties of pesticides, namely the polarity of the analyzed pesticides, it has led to different sample preparation methods having advantages and disadvantages. For example, the QuEChERS method has become popular due to the minimal use of traditional analytical stages, solvents, and glassware [20,21,30].

The usual procedure for analyzing a large amount of grape pesticide residues uses acetonitrile [10,15,22,29,36] as the organic solvent. One of the disadvantages of solvent extraction is the loss of essential pesticides in acidic crops such as grapes.

The extraction solvents used for SLE in specific methods for grapes demonstrate higher versatility [38]. In addition to acetonitrile, other organic solvents were also used, such as acetone [17,24,26] and methanol [24,39]. One of the reasons for such high variability of extraction solvents maybe that special methods have been developed and optimized for a small group of pesticides (often from the same chemical family and analyzed by the same method).

## 3. Instrumental Detection Method

**Figure 4:**
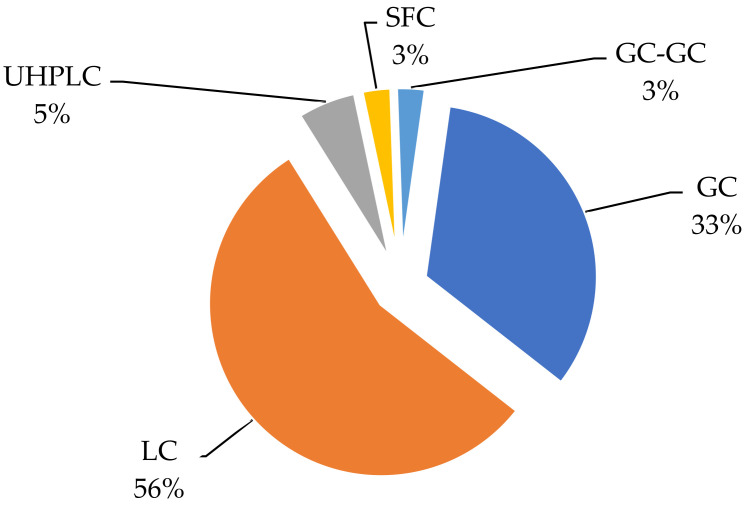
The most used detection methods in the separation of pesticides in grapes.

Due to the interference of different matrices, it becomes difficult to understand the method of determining pesticides in real samples. In recent years, the most used strategies for detecting and quantifying pesticides in grapes have been gas and liquid chromatography due to their sensitivity, separation ability, and identification. In addition to these methods, others were also used to determine pesticide residues in real grape samples Table 3. The data show that many analytical methods are used to analyze pesticide residues in grape samples, Figure 4.

**Figure 5:**
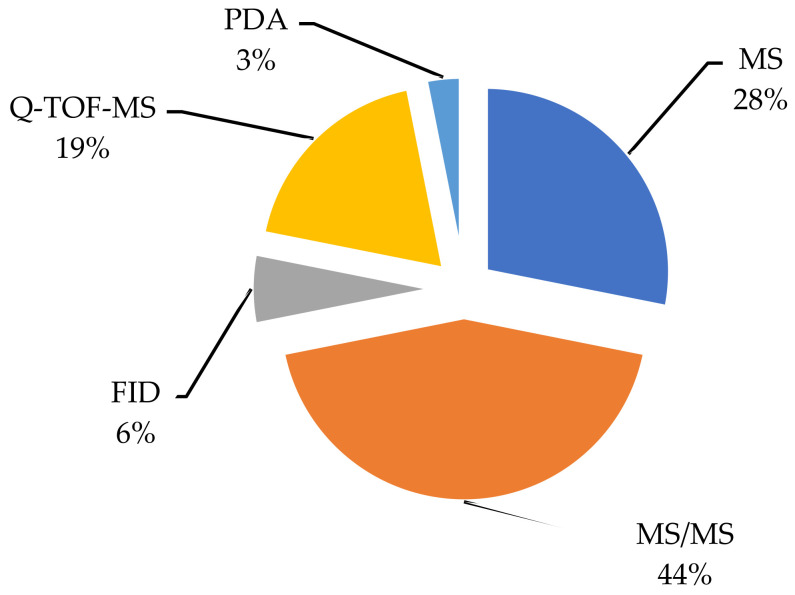
Commonly used detectors in determining the residual amounts of pesticides in grapes.

The modern method of separating and identifying residual amounts of pesticides in grapes also requires a good foundation in the form of detectors. MS is a very sensitive analyzer [33,50] to determine organophosphate pesticides, but this does not exclude the fact that it remains an excellent analyzer for other classes of pesticides. Photodiode array detector (PDA) remains a specific analyzer [36] to determine peritroidal pesticides in grapes. Analyzers such as (Q-TOF-MS, and MS/MS) are more often used in specific methods [14,16,18,22,25,26,27] according to the definition of numerous pesticide residues in grapes. Statistics on the most frequently used analyzer for 2015–2021 are given in Figure 5.

### 3.1. Gas Chromatography

Due to the presence of matrix interference, it becomes difficult to create a method for determining pesticides. In recent years, GC and LC have been particularly frequently used strategies for the detection and quantification of pesticides in fruits and vegetables due to their sensitivity, separation, and identification ability. In addition, other methods were also used to determine pesticide residues, such as the determination of organophosphate pesticides in food by the colorimetric method [51].

Most published studies claim that the detection of pesticides was carried out using GC in combination with various detectors. Because of their sensitivity, detectors such as the MS/MS detector [10,23], MS [50], and flame ionization detector (FID) [26] are used. In addition, mass detection methods are also used to increase the sensitivity of the method, which are equipped with analyzers such as time of flight (TOF) [52].

GC-MS/MS results showed that the influence of the matrix on the method was insignificant. The authors concluded that the method could be used for real samples [10,25].

As an alternative to a quadrupole mass analyzer, an ion trap (IT) was also used, in which the scanning mode allows you to control the selection of ions after collection [53].

In gas chromatography, the following columns were most often used to determine pesticides in grapes: SLB 146-5ms fused silica, HP-5 capillary column, DB5, TM-1 fused silica [10,15,23,26,50].

However, over the last decade, the use of GC methods has declined due to the more extensive use of polar pesticides (less resistant and highly toxic), which are considered unsuitable for GC detection methods due to their volatility and poor heat resistance.

### 3.2. Liquid Chromatography

The extract was filtered and diluted before being introduced into ultra-efficient liquid chromatography connected to tandem mass spectrometry with an electrospray ionization source (ESI) in positive and negative modes [9,26,36]. The authors of [26] reported a method for detecting traces of pyrethroid residues in plant matrices using the extraction method of magnetic nanoparticles coated with polystyrene, in combination with the method of high-performance liquid chromatography HPLC using PDA.

In some studies, with a liquid chromatograph, such analyzers as a triple quadrupole were used. Such an analyzer provides very high sensitivity and high separation capability relative to other alternative analyzers. Moreover, LC-MS/MS optimization provides shorter execution time with high specificity and increased sensitivity [19,43,54]. The authors tested the effectiveness of HPLC for the detection of foxime in grapes [40].

Liquid chromatography with a mass spectrometric detector (LC-MS/MS) was used to determine pesticides in various matrices, including grapes. Methods of rapid multi-analysis of metalaxyl-M, boscalid, fluopicolide, and its metabolites in wine, grapes, and raisins were created. The pesticides used for the analysis showed good linearity. The method is suitable because it provides a basis for the simultaneous determination of target pesticides in grapes [16,17].

**Table 3:** Detection methods for assessing pesticide residues in fruits and vegetables.

№	Detection Method	Number/Name of Analytes	LODs (mg/kg)	LOQs (mg/kg)	Reference
1	FI-MS/MS	1 pesticide			[38]
2	GC/MS-MS	8 pyrethroid pesticides	0.02–0.5		[25]
3	GC-GC/TOF-MS	5 organophosphorus pesticides		0.001–0.01	[34]
4	GC-MS	2 organophosphorus pesticides	0.02–0.30	0.07–1.0	[50]
5	GC-MSHPLC-MS-MS	48 pesticides		2.90–7.050.31–5.15	[18]
6	GC-MSGC-FID	7 multiclass pesticides	0.34–1.2	1.1–4.0	[15]
7	GC-MS	6 organophosphorus pesticides	0.04–10	0.4–35	[33]
8	GC–MSGC-FID	9 multiclass pesticides	0.34–1.2	1.1–4.0	[26]
9	GC-MS/MS	5 multiclass pesticides			[10]
10	GC-MS/MS	6 multiclass pesticides	3	<10	[23]
11	GC-MSD	6 multiclass pesticides			[24]
12	GC–Q-TOF-MSLC–Q-TOF-MS	733 pesticide multi-residues	10		[52]
13	HPLC	11 fungicides			[20]
14	HPLC	6 triazole fungicides	0.022–0.071		[35]
15	HPLC	2 multiclass pesticides	0.26–0.0039	<0.001	[31]
16	HPLC	2 organophosphate pesticides	1.2–4.2		[32]
17	HPLC	5 multiclass pesticides	0.02–0.0392	0.072–0.128	[36]
18	HPLC-MS	Phoxim			[40]
19	HPLC-MS/MS	7 multiclass pesticides	0.0002–0.005	0.001–0.01	[16]
20	HPLC-PDA	5 pyrethroid pesticides	0.02–0.039	0.072–0.128	[36]
21	LC-MS	14 fungicides	0.002–0.01	0.01	[19]
22	LC-MS	7 multiclass pesticides			[37]
23	LC-MS/MS	96 multiclass pesticides	0.01–5.86		[9]
24	LC-MS/MS	5 multiclass pesticides	0.007–0.01		[28]
25	LC-MS/MS	5 multiclass pesticides			[13]
26	LC-MS/MS	3 multiclass pesticides	2.1–8.7	<0.1	[21]
27	LC-MS/MS	2 multiclass pesticides			[30]
28	LC-MS/MS	49 fungicide and pesticides		0.2–13	[39]
29	LC-MS-MS	136 pesticides		0.5–10 ng/g	[17]
30	RP-HPLC	3 multiclass pesticides			[29]
31	SFC-Q-TOF/MS	Diniconazole	0.010–1.0	0.005	[37]
32	UHPLC/TOF-MS	60 multiclass pesticides	0.3–3.8	0.8–11.8	[22]
33	UHPLC-MS/MS	250 pesticides		0.6–6.0	[14]
34	UPLC-Q-TOF-MS	134 pesticides		<10	[55]

Gas and liquid chromatography are common methods for quantitatively determining pesticide residues in grapes and include different detectors, such as nitrogen phosphate detector (NPD), photometric flame detector (FPD), and fluorescence detector. However, these methods are used to determine the residues of pesticides of the same type [52]. In addition, the number of target analytes is not large, and the sensitivity usually cannot match the level of microelement detection. The search for methods of multiclass analysis of pesticides in recent years has attracted much attention. The increased sensitivity and resolution of instruments that perform full-scan analyses have allowed the development of new methods based on liquid chromatography, giving this method wide use [9,17,28,37].

Q-TOF-MS is a modern detector that is not used so often due to its high cost. This detector, paired with gas and liquid chromatography, provides a promising prospect for use in non-targeted screening and quantitative determination of multiple pesticide residues in grapes. Q-TOF-MS was found to be reliable for confirming pesticide residues in grape [25,27,48,52]. According to the above articles, this detector provides a wide range of screening, provides accurate quantitative determination of target compounds, has good adaptability, and high sensitivity, and can be used to increase the number of detected pesticides and detection capabilities compared to chromatography methods. Due to its many detectors characteristics (TOF)-MS, mass spectrometry (MS) could detect a wide range of compounds before its development and rapid spread [55]. Q-TOF-MS is an excellent detector with a great future, but traditional detectors such as MS are used by scientists from different countries [15,18,19,50].

**Figure 6:**
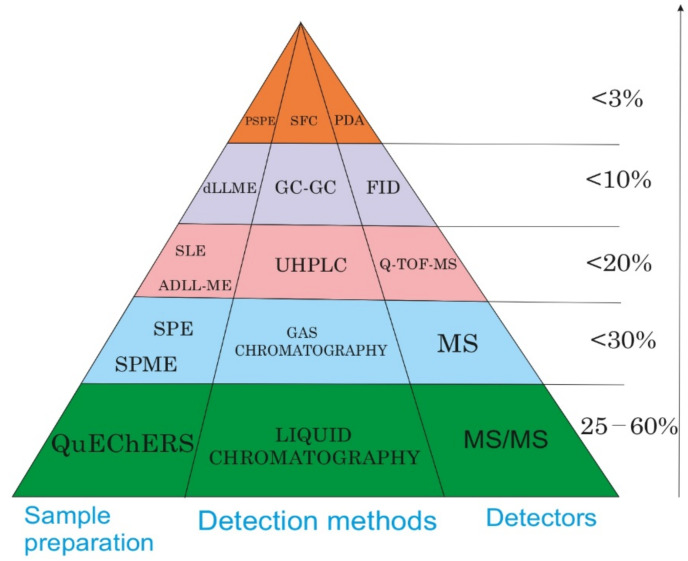
Frequently used analyzers, sample preparation methods, and methods for determining the residual amounts of pesticides in grapes were made based on this review for 2015–2021.

A review was conducted for 2015–2021 on sample preparation methods and the determination of residual pesticides of different classes in grapes Figure 6. According to the survey data and the pyramid, over the past five years, the methods of sample preparation and determination of pesticides in grapes have been sufficiently changed or replaced with others compared to other years. As can be seen, QuEChERS is the most popular method of sample preparation; LC-MS/MS is a popular method of separating and determining pesticides in grapes. Thus, it can be concluded that despite the specific characteristics of grapes, many countries are engaged in the study of this product because of its beneficial properties. So far, the question remains on how to improve and create new methods of sample preparation and separation, and determination of pesticides in grapes, to control the conversion of permissible concentrations.

## 4. Conclusions

Many studies have been published in recent years to assess the residual quantities of pesticides in grapes. Currently, most equipment used in pesticide residues analysis requires large sample volumes, high prices, different organic solvents, and long analysis time. Acetonitrile, ethyl acetate, and acetone are used as a solvent in several works. On the other hand, the review shows that QuEChERS, SPME and SPE are the most prevalent methods of sample preparation used in grape analysis. SPME is a green sample preparation method and, as such, is not used in any organic solvents. LC and GC are common analytical separation procedures, and they are frequently combined with MS and MS/MS as QqQ for extremely sensitive identification and quantification. GC coupled with MS is a convenient analytical tool for pesticides analysis in grapes because of it is fast detection, high separation efficiency and ease of operation. Detectors such as MS/MS and QqQ are the most sensitive and can identify low concentrations of pesticides; however, due to their high cost, the use of these detectors is limited.

Currently, there is no widely accepted method for assessing pesticide residues in grapes. This is a challenging task due to the large number of pesticides from diverse chemical classes, as well as the fact that these analytical approaches should apply in several countries with different opportunities. The future development of analytical methods requires enabling the rapid, sensitive, cheaper and easy to use analysis of pesticides in grape.

## Figures and Tables

**Table 2:** Summarizing the extraction and pretreatment method for assessing pesticide residues in grapes 2015–2021 (Database Scopus, Web of Science).

№	Extraction Method	Matrix	Number/Name of Analytes	Recovery (%)	Study Region, Country	Reference
1	Solid-phase extraction (SPE)	Grape, brinjal, cabbage, cauliflower, guava, okra, onion, potato, apple, banana, mango, orange, and pomegranate	60 multiclass pesticides	74–111	India	[22]
2		Berry fruits, raspberry, strawberry, blueberry, and grape	5 multiclass pesticides	63–137	China	[36]
3		Grape, cauliflower, and leek	2 pyrethroid pesticides	88.5–94.2	China	[32]
4		Table grape	3 multiclass pesticides	90.55–105.40	Republic of Macedonia	[29]
5		Fruit juice (grape, sour cherry, peach, apple, orange, apricot, and mango)	7 multiclass pesticides	87–107	Tabriz, Iran	[15]
6		Grape	7 multiclass pesticides	90–104	Germany	[37]
7	Dispersive liquid-liquid microextraction (dLLME)	Mango, apricot, peach, apple, and grape	9 multiclass pesticides	46–95	Karaj Iran	[26]
8	Solid–liquid extraction (SLE)	Chickpeas, apples, and grapes	Glyphosate	60–111	Italy	[38]
9	Assisted dispersive liquid-liquid microextraction method (ADLL-ME)	Vineyard soils, grapes	6 multiclass pesticides	75–100	Spain	[24]
10	Solid-phase microextraction (SPME)	Vineyard soils, grapes	49 multiclass fungicides and insecticides	70–130	Spain	
11		Apples, blueberries, strawberries, and grapes	136 pesticides	-	Canada	[17]
12		Grapes	8 pyrethroid pesticides	80.9–104.6	China	[25]
13		Grape	6 organophosphorus pesticides	87.5–112	Iraq	[33]
14		Grape	5 organophosphorus pesticides	-	Canada	[34]
15	Pressurized liquid extraction (PLE)	Grapes and grape juice	12 fungicides	70–130	Spain	[39]
16	Polymeric solid phase extraction (PSPE)	Grape	5 multiclass pesticides	-	Iran	[10]
17	Quick, easy, cheap, effective, rugged, and safe method (QuEChERS)	Grape	2 multiclass pesticides	31.7–54	China	[31]
18		Grape	2 multiclass pesticides	76.88–97.05	China	[30]
19		Table grape	3 multiclass pesticides	83.2–105.4	China	[21]
20		11 vegetable samples (lettuce garlic shoot, yam, celery, carrot, pepper, chives, cowpea, tomato, spinach, cabbage, apple, kiwi, pear, grape)	11 multiclass pesticides	71.3–116.7	China	[20]
21		Grape	250 pesticides	70–120	Spain	[14]
22		Rape and grape	5 multiclass pesticides	14.7–59.8 (Rape)72.1–100 (Grape)	China	[13]
23		Grape or soil sample	5 multiclass pesticides	71.6–107.7	China	[28]
24		Grapes, wine, and raisins	7 multiclass pesticides	78.8–106.3	China	[16]
25		Grape and grape juice	6 multiclass pesticides	74–101	India	[23]
26		Table grape	48 pesticides	51–127	Turkish	[18]
27		Grape	Phoxim	73.60	China	[40]
28		Grape	Diniconazole	69.8–102.1	China, USA	[27]

## Data Availability

No data were provided in the study.

## Abbreviations

AChE	enzyme acetylcholinesterase
ADLL-ME	assisted dispersive liquid-liquid microextraction method
dLLME	dispersive liquid-liquid microextraction
DSPE	dispersive solid-phase extraction
EC	European Commission
GC	gas chromatography
HPLC	high performance liquid chromatography
LAC	limits of acceptable concentrations
LC	liquid chromatography
MIP	molecular imprinted polymer
MSPD	matrix solid phase dispersion
OPPs	organophosphorus compounds
PDMS	polydimethylsiloxane
PLE	pressurized liquid extraction
PSA	primary secondary amine
PSPE	polymeric solid phase extraction
QuEChERS	Quick, Easy, Cheap, Effective, Rugged, and Safe
RSD	Relative Standard Deviation
SLE	solid–liquid extraction
SPE	solid-phase extraction
SPME	solid-phase microextraction
PDA	diode-array detection
